# The Cytohesin Coiled-Coil Domain Interacts with Threonine 276 to Control Membrane Association

**DOI:** 10.1371/journal.pone.0082084

**Published:** 2013-11-26

**Authors:** Kevin G. Hiester, Lorraine C. Santy

**Affiliations:** Department of Biochemistry and Molecular Biology, Pennsylvania State University, University Park, Pennsylvania, United States of America; University of Illinois at Chicago, United States of America

## Abstract

Cell migration is regulated by a number of small GTPases, including members of the Arf family. Cytohesins, a family of Arf-activating proteins, have been extensively implicated in the regulation of Arfs during migration and cell shape change. Membrane association of both the Arf and its activating protein is a prerequisite for Arf activation. Therefore regulating the extent of cytohesin membrane association is a mechanism for controlling the initiation of cell movement. We have discovered a novel intramolecular interaction that controls the association of cytohesins with membranes. The presence of the coiled-coil domain reduces the association of cytohesin 2 with membranes. We demonstrate that this domain interacts with more C-terminal regions of the protein. This interaction is independent of another previously identified autoinhibitory conformation. A threonine residue (T276) in the cytohesin 2 PH domain is a target for phosphorylation by Akt. Mutation of this threonine to aspartic acid, to mimic phosphorylation, disrupts the binding of the coiled-coil domain to c-terminal regions and promotes membrane association of cytohesin 2. The presence of a second autoinhibitory interaction in the cytohesins suggests that these proteins can act a signal integrators that stimulate migration only after receive multiple pro-migratory signals.

## Introduction

Cell migration requires precisely coordinated changes in cell shape and polarity. Additionally, within an organism the initiation of migration is a tightly regulated process. Aberrant migration underlies pathological processes such as metastasis, while the failure to properly initiate migration can lead to immune disfunction and poor wound healing. The cell shape and polarity changes that underlie cell movement are regulated by a number of small GTPases, including members of the Ras, Rho and Arf families [1]. Small GTPases act as switches that convert between a GDP-bound “off” state, and a GTP-bound “on” state. The interconversion of GTPases between the on and off states requires accessory proteins. GTPases are turned on by guanine nucleotide exchange factors (GEFs), and they are turned off by GTPase activating proteins (GAPs) [2].

**Figure 1:**
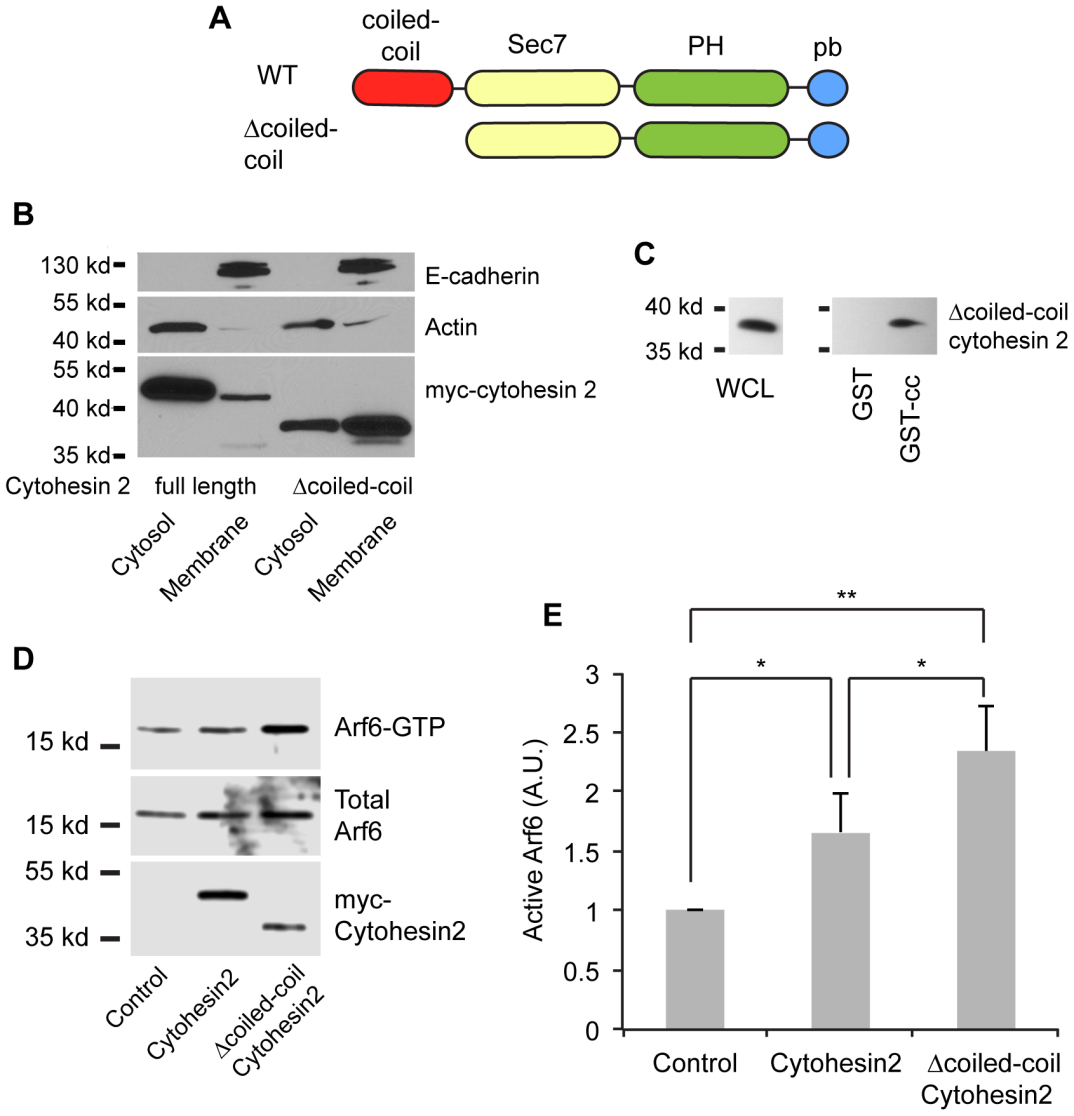
The cytohesin 2 coiled-coil domain binds the remainder of the protein and impairs membrane localization and Arf6 activation. A) Domain organization of full-length cytohesin 2 and Δcoiled-coil cytohesin 2. The arrangement of the coiled-coil, Sec7, pleckstrin homology (PH) and polybasic (pb) domains is shown. B) The coiled-coil domain of cytohesin 2 impairs membrane localization. MDCK cells were infected with adenovirus encoding cytohesin 2 or Δcoiled-coil cytohesin 2 for 4 hours and then fractionated into cytosol and total membranes. Fractions were Western blotted with 9e10 mouse anti-myc to detect the cytohesins and with antibodies to detect E-cadherin (membrane marker) and actin (cytosol marker). C) The cytohesin coiled-coil domain binds to the remainder of the protein. 293 cells were transfected with constructs encoding Δcoiled-coil cytohesin 2, lysed, and the cleared lysate incubated with GST or GST-coiled-coil bound to glutathione beads. Δcoiled-coil cytohesin 2 in the lysate and bound to the beads was detected by Western blotting with mouse anti-myc. D, E) Δcoiled-coil cytohesin 2 is a more active GEF than WT. MDCK cells were infected with adenovirus encoding cytohesin 2, Δcoiled-coil cytohesin 2 or virus in the presence of 20 ng/ml doxycycline to prevent transgene expression. After 3 hours of expression Arf6-GTP was isolated by pulldown with GST-GGA3 and quantitated by Western Blot. D) Representative gel from the experiments quantified in E. E) Levels of Arf6 activation in 8 pulldown experiments were quantified. Data shown are mean ± standard error. The levels of active Arf6 in the various samples were compared using a paired T-test (N = 8). ** = p<0.01, * = p<0.02.

Arf GTPases were first identified as regulators of vesicular trafficking. More recently Arfs, Arf6 in particular, have been shown to regulate the cortical actin cytoskeleton, cell shape and migration. Mammalian cells express 6 different Arf proteins and more than 15 different Arf GEFs [3], [4]. A majority of these GEFs can activate Arf6. Different GEFs are thought to acting at different subcellular locations and during different Arf6 regulated processes. One family of Arf-GEFs, the cytohesins, has been extensively implicated in the regulation of cell shape, adhesion and migration. Cytohesin family members share a conserved domain architecture consisting of an n-terminal coiled-coil domain, a Sec7 catalytic domain, a pleckstrin homology (PH) domain, and a c-terminal polybasic domain (figure 1A) [3].

The initial step in Arf activation is the rearrangement of an n-terminal helix and the insertion of a myristol group attached to this helix into a membrane bilayer [5], [6]. Therefore Arf activation occurs only at membrane surfaces. The cytohesins bind to membranes by the combined actions of their PH and polybasic domains. The PH domain binds to phosphoinositides and the polybasic domain binds to negatively charged phospholipid headgroups [7].

We have previously demonstrated that overexpression of cytohesin 2/ARNO potently stimulates migration [8], [9]. Therefore cytohesin activity needs to be tightly regulated in order to prevent inappropriate cell migration. A structural study of the cytohesins demonstrated the presence of an autoinhibitory interaction in these proteins [10]. The linker region between the Sec7 and PH domains and the c-terminal polybasic domain form a psuedostubstrate that inhibits the catalytic Sec7 domain. This inhibitory conformation is relieved either by phosphorylation of residues within the polybasic domain, or by binding of active Arfs to the PH domain [10]. The ability of active Arfs to relieve autoinhibition of the cytohesin Arf-GEFs produces a positive feedback loop leading to robust Arf activation [11].

The crystal structure that lead to the identification of the psuedosubstrate autoinhibitory conformation contained the Sec7, PH and pb domains of cytohesin 3 [10]. Importantly it did not contain the n-terminal coiled-coil domain. We have now determined that the cytohesin coiled-coil domain participates in a second, independent autoinhibitory interaction. The cytohesin coiled-coil domain binds to the rest of the protein and reduces its association with membranes. A recent study demonstrated that phosphorylation of a threonine in the cytohesin PH domain promotes membrane binding by an unknown mechanism [12]. We find that mutation of this residue (T276 in cytohesin 2) to a phosphmemetic aspartic acid disrupts the interaction of the coiled-coil domain with the rest of the protein and promotes the recruitment of cytohesin 2 to membrane surfaces. These data suggest that full activation of the cytohesin Arf GEFs may require the integration of two separate signals to relieve two independent auto-inhibitory interactions.

## Materials and Methods

*Antibodies and Reagents*. The 9e10 antibody against myc and mouse anti-HA (16B12) were purchased from Covance (Princeton, NJ). Mouse anti E-cadherin, and mouse anti-actin were obtained from BD (San Jose, CA). Mouse anti-green fluorescent protein (GFP; B-2) was purchased from Santa Cruz Biotechnology (Santa Cruz, CA). Mouse anti-mCherry was obtained from Novus Biologicals (Littleton, CO). Glutathione resin was obtained from Thermo-Fisher. Secondary antibodies were obtained from Jackson ImmunoResearch (West Grove, PA). Western blots were developed using Millipore ECL substrate and visualized by exposure to film, or with the cDigit imaging system from LiCor (Lincoln, NE). Recombinant Akt1 was purchased from Millipore (Billerica, MA). Rabbit monoclonal antibody (110B7E) for detecting phosphorylated Akt substrates was obtained from Cell Signaling (Danvers, MA).

*Cell Lines*. Tet-off MDCK cells were obtained from Clontech (Mountain View, CA) and 293H cells were obtained from Invitrogen (Carlsbad, CA). Both cell lines were maintained in DMEM plus 10% FBS and penicillin, streptomycin and fungizone. All cells were maintained at 37°C and 5% CO2. FBS was obtained from Gemini (West Sacramento, CA). MDCK cells were transfected using the Neon transfection system (Invitrogen) with the settings (1400, 20, 2). 293 cells were transfected using calcium phosphate transfection or using Lipofectamine 2000 according to the manufacturer's instructions.

*Expression constructs*. Recombinant adenoviruses and expression vectors encoding full-length cytohesin 2/ARNO, Δcoiled-coil cytohesin2, GRASP, IPCEF and GST-coiled-coil have been previously described [8], [9]. Point mutants were introduced using a two-step mutagenesis protocol [13] and confirmed by sequencing. GST fusions of the cytohesin 2 PH domain were created by amplifying the PH domain (amino acids 250–380) from WT or T276A cytohesin 2 and fusing it in frame into pGEX2Tk.

*Cell fractionation*. Fractionation of MDCK cells into cytosol and total membranes was performed as previously described [9].

*GST pulldowns*. GST and GST-coiled-coil were produced as previously described [9]. MDCK or 293 cells expressing the indicated proteins were lysed in 50 mM Tris pH 7.5, 100 mM NaCl, 2 mM MgCl2, 1% NP-40, 10% glycerol, 0.1 mM PMSF and 1 µg/ml peptatin, leupeptin and antipain. Unsolubilized material was removed by centrifugation at 15,000× g for 10 minutes at 4°C. An aliquot of the cleared whole cell lysate was saved and the remainder was split and incubated with GST and GST-cc bound to glutathione beads at 4°C rotating as previously described [9]. After binding the beads were washed three times with lysis buffer and eluted into SDS-PAGE sample buffer.

*Arf Activation Assays*. MDCK cells were transfected with pTRE2, pTRE2-cytohesin 2 or pTRE2-T276A cytohesin 2 using the Neon system. The cells were incubated in the presence of 5–7 ng/ml doxycycline for 2.5 hours to allow them to attach. Then the doxycycline was removed and the cells incubated for 1.5 more hours to allow cytohesin 2 expression. Alternatively, they were infected with adenovirus encoding cytohesin 2, Δ coiled-coil cytohesin 2, or adenovirus encoding cytohesin 2 in the presence of 20 ng/ml doxycycline to repress transgene expression for 3 hours. The level of active Arf6 was then determined by pulldown with GST-GGA3 as previously described [14].

*Akt phosphorylation*. GST fusions to the PH domain of WT or T276A cytohesin 2 were purified as previously described [9] and eluted from the glutathione beads by incubation in 50 mM Tris pH 8.0, 10 mM Glutathione. Akt1 dependent phosphorylation was tested by incubating 5 µg of each protein for 30 minutes at 37°C in the presence or absence of 450 ng of recombinant Akt1 in 25 mM Tris pH 7.5, 10 mM MgCl_2_, 5 mM β-glycerophosphate, 0.1 mM Na_3_VO_4_, 2 mM DTT, 20 µM ATP. Phosphorylation by Akt was revealed by Western blotting with an anti-Akt substrate antibody,

## Results

Arf-GEFs must be at a membrane surface to activate Arfs [5], [6]. Therefore regulation of the membrane recruitment of Arf-GEFs can be a powerful mechanism to control the activation of Arf family members. Intriguingly, we noticed during membrane fractionation of cytohesin mutants that a cytohesin truncation mutant lacking the coiled-coil domain (Δcoiled-coil cytohesin 2) was consistently more prevalent in membrane fractions than the full-length protein. As is shown in figure 1B a larger fraction of the Δcoiled-coil is present in the total membrane fraction when compared to the wild-type protein. This is unexpected as both proteins contain the PH and polybasic domains, which cooperate to bind cytohesins to the cytoplasmic leaflet of membranes [7]. Although multiple crystal structures of the cytohesins have been solved, none of them contain the coiled-coil domain so the structural relationship of the coiled-coil domain to the rest of the protein is unknown [10], [15]–[17]. We hypothesized that the cytohesin coiled-coil domain might be binding to the rest of the protein and blocking it from binding to membranes. To test this hypothesis we tested the ability of the Δcoiled-coil cytohesin 2 mutant to bind to the coiled-coil domain *in trans*. Glutathione sepharose beads and GST or a fusion of GST to the cytohesin coiled-coil domain were incubated with a cleared cell lysate expressing myc-tagged Δcoiled-coil cytohesin 2. The Δcoiled-coil cytohesin 2 bound to the GST-coiled-coil but not to GST alone (figure 1C). This supports the conclusion that the coiled-coil domain binds to the rest of the protein. Therefore the coiled-coil domain may act as an auto-inhibitory domain and block the cytohesin membrane binding domains. If this is the case then the full-length protein should have less GEF activity than the truncated version. We used pulldown assays to compare the ability of WT and Δcoiled-coil cytohesin 2 to activate Arf6. When expressed at similar levels Δcoiled-coil cytohesin 2 activated Arf6 more than did the full-length protein (figure 1 D, E). These data support the conclusion that the coiled-coil domain of cytohesin 2 regulates its activity by modulating its recruitment to membrane surfaces.

**Figure 2:**
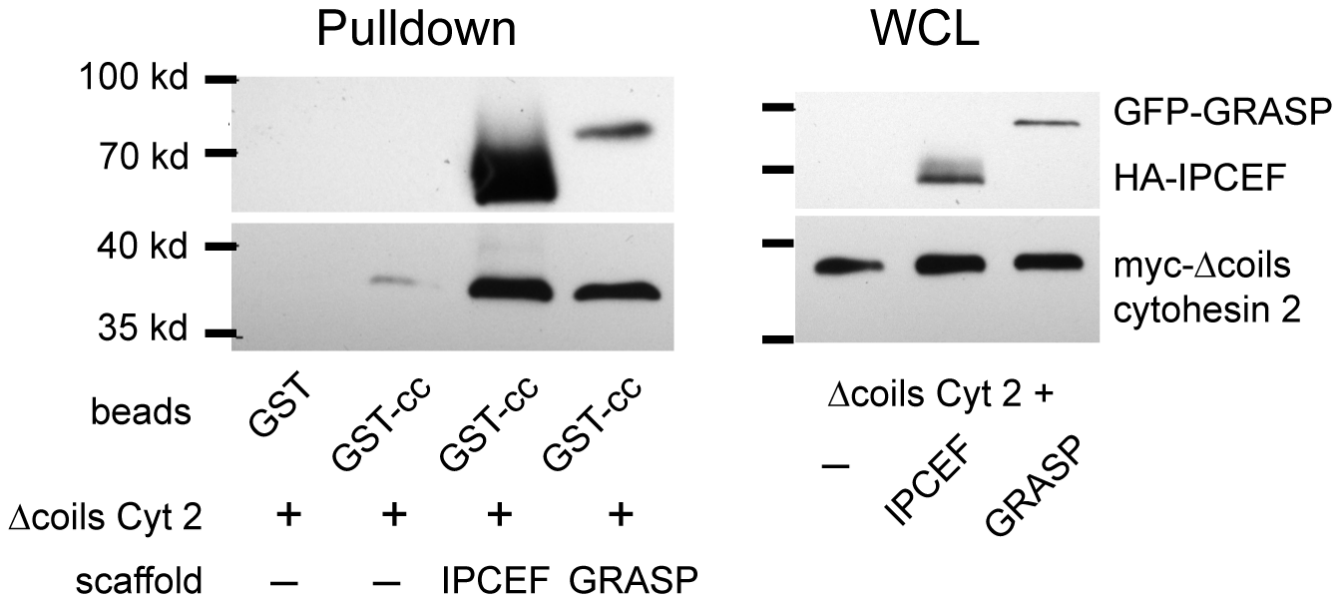
Scaffolding proteins that bind coiled-coil domain do not disrupt the intramolecular interaction. MDCK cells were infected with adenoviruses encoding the indicated proteins. The cells were lysed and the lysates incubated with GST-coiled-coil as described above. The lysates and bound proteins were Western blotted with mouse anti-GFP, mouse anti-HA and mouse anti-myc.

If the coiled-coil domain were an auto-inhibitory domain, then its interaction with the rest of the protein would have to be disrupted for membrane binding and Arf-activation to occur. Several scaffolding proteins, such as CNK3/IPCEF and GRASP, have been identified that bind to the cytohesin coiled-coil domain [14], [18], [19]. At least one of these proteins has been reported to translocate from the cytosol to the plasma membrane with cytohesin 2 in response to serum stimulation and to promote Arf activation [19]. We therefore tested if binding of these scaffolds to the cytohesin coiled-coil domain prevents binding of this domain to the rest of cytohesin 2. GST-coiled-coil was incubated with a cell lysate expressing Δcoiled-coil cytohesin 2 and either IPCEF or GRASP. Although these scaffold proteins bound to the GST-coiled-coil they did not prevent Δcoiled-coil cytohesin 2 from also binding (figure 2). In fact in many cases the presence of these scaffolds seemed to enhance the association of Δcoiled-coil with GST-coiled-coil. Therefore rather than disrupting the auto-inhibitory interaction of the coiled-coil domain with the rest of the protein, these scaffolds may stabilize the cytohesin in this inactive conformation until other signals disrupt it.

**Figure 3:**
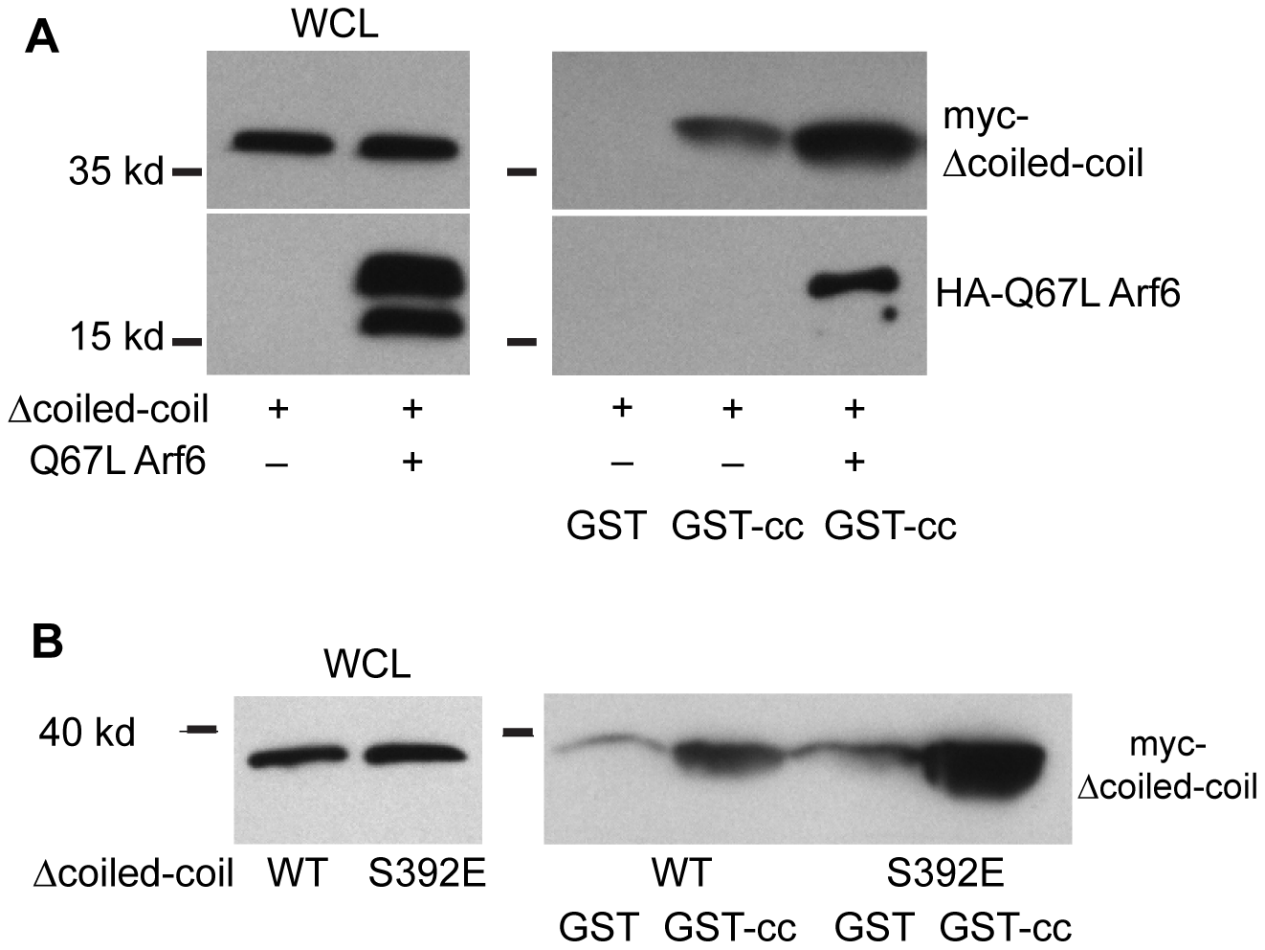
Disruption of the cytohesin pseudosubstrate inhibitory interaction does not disrupt the coiled-coil intramolecular interaction. A) MDCK cells were infected with adenovirus encoding Δcoiled-coil cytohesin 2 and adenovirus encoding HA-tagged ARF6 Q67L as indicated. The cells were lysed and half of each lysate incubated with GST or GST-coiled-coil. Lysates and bound proteins were Western blotted with mouse anti-myc and mouse anti-HA. B) 293 cells were transfected with wild-type or S392E Δcoiled-coil cytohesin 2. The cells were lysed and half of each lysate incubated with GST or GST-coiled-coil. Lysates and bound proteins were Western blotted with mouse anti-myc to detect the cytohesins.

Another auto-inhibitory conformation of the cytohesins has been described. The linker region between the Sec7 and PH domains in conjunction with the C-terminal polybasic domain, acts as a pseudosubstrate to inhibit the catalytic Sec7 domain [10]. This auto-inhibitory interaction is relieved by binding of active Arf family members to the cytohesin PH domain or by PKC-dependent phosphorylation of residues within the polybasic domain [10]. We tested whether the coiled-coil domain might bind to this auto-inhibitory conformation to provide an additional layer of negative regulation by determining if disrupting the psuedosubstrate conformation prevents binding of the coiled-coil domain to the rest of the protein. The Arf family members, Arf6 and Arl4, can bind to cytohesin PH domains when they are in the GTP-bound state [20]–[22]. This interaction promotes recruitment of the cytohesin to membranes [20]–[22]. Subsequently this interaction was shown to disrupt the cytohesin psuedosubstrate autoinihibitory conformation [10]. Therefore we tested if co-expression of Arf6 Q67L, a GTP-locked mutant, would disrupt the interaction of the cytohesin coiled-coil domain with the rest of the protein. Rather than disrupting this interaction, co-expression of Arf6 Q67L with Δcoiled-coil cytohesin 2 seemed to promote this interaction (figure 3A). In fact we could also detect Arf6 Q67L bound to the GST-coiled-coil beads (figure 3A). Since Arf6 Q67L binds to the cytohesin PH domain not to the coiled-coil domain, it must be binding to the beads as part of a tertiary complex containing the GST-coiled-coil, Δcoiled-coil cytohesin 2, and Arf6 Q67L. The existence of such a complex confirms that Arf6 Q67L cannot be disrupting the interaction of the coiled-coil domain with the rest of the protein.

The second mechanism that can disrupt the psuedosubstrate auto-inhibitory interaction is the PKC dependent phosphorylation of residues in the cytohesin polybasic domain [10]. The polybasic domain residue in cytohesin 2 that is phosphorylated by PKC is serine 392 [23]. We mutated this serine to glutamic acid (S392E) to mimic phosphorylation and tested if this mutation prevented the interaction of Δcoiled-coil cytohesin 2 with the GST-coiled-coil. Similar to what we saw with Arf6 Q67L, we found that Δcoiled-coil S392E cytohesin 2 consistently bound to GST-coiled-coil better than the Δcoiled-coil cytohesin 2 (figure 3B). These data suggest that the coiled-coil domain preferentially binds to the conformation where the pseudo-substrate inhibition has been relieved. Therefore a second signal would be needed to disrupt the interaction of the coiled-coil domain with the rest of the protein and to allow membrane recruitment and Arf activation.

**Figure 4:**
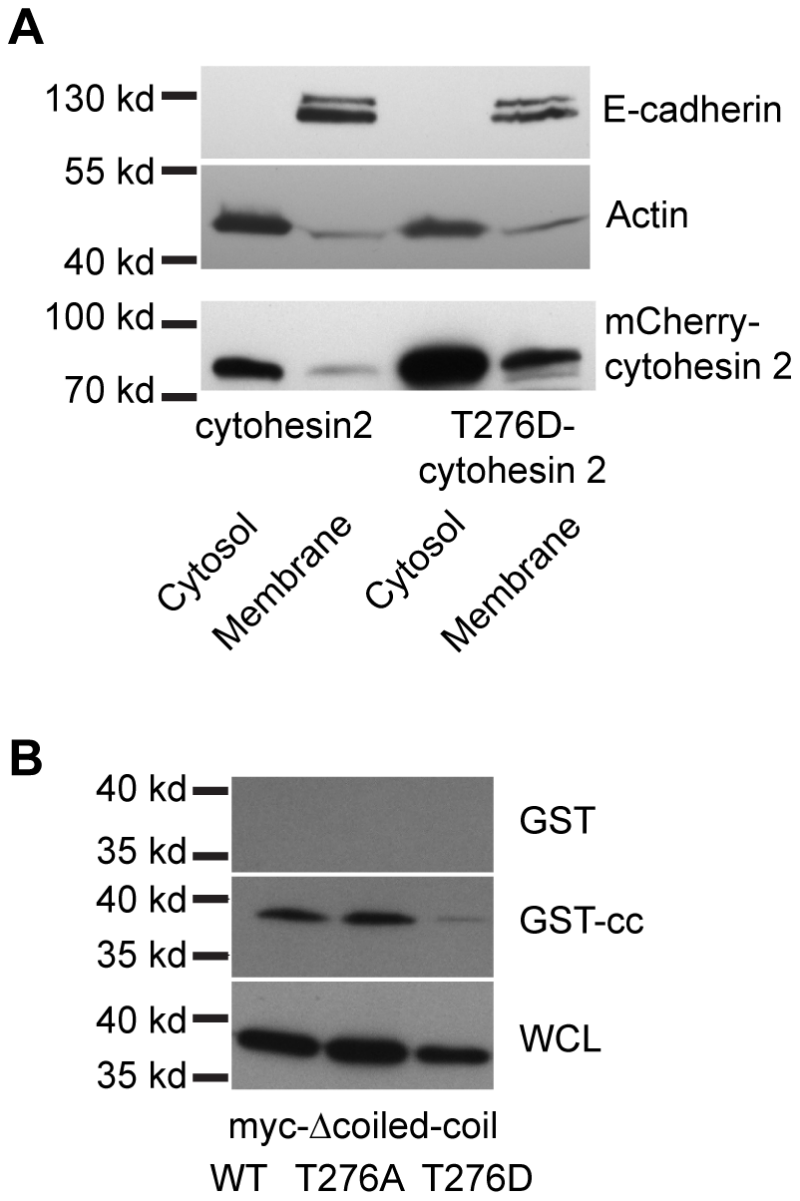
Threonine 276 is required for the intramolecular interaction, and for inhibition of membrane binding. A) Mutation of threonine 276 to aspartic acid promotes the association of cytohesin 2 with membranes. MDCK cells were transfected with constructs encoding mCherry-tagged wild-type or T276D cytohesin 2 and fractionated into cytosol and total membranes. The fractions were Western blotted with mouse anti-mCherry, mouse anti-E-cadherin and mouse anti-actin. B) Mutation of T276 to aspartic acid disrupts the interaction of the coiled-coil domain and the remainder of the protein. 293 cells were transfected with the indicated cytohesin mutants, lysed and half of each lysate incubated with GST or GST-coiled-coil for 2 hours. Expression of cytohesins and bound cytohesins were detected by Western blotting with mouse anti-myc.

A recent report determined that cytohesin 3 activates Arf6 and stimulates Glut4 exocytosis in response to insulin signaling. This study identified two residues in cytohesin 3 that are phosphorylated by Akt downstream of insulin and promote this trafficking step. Phosphorylation of one site, S155, in the Sec7 domain stimulates the catalytic GEF activity of the Sec7 domain. Phosphorylation of the second residue, T280, promoted membrane recruitment through an unknown mechanism [12]. This threonine residue is conserved in cytohesin 2, T276. It is located within the cytohesin PH domain close to the phosphoinositide binding site. It is just 4 amino acid residues away from a key glycine motif that determines the phosphoinositide affinity of the cytohesin PH domains [16], [24], [25]. We hypothesized that if the coiled-coil domain of cytohesin 2 interacts with the PH domain to prevent membrane binding, then it could be binding this threonine residue. Phosphorylation of this residue would disrupt this interaction and allow binding of cytohesins to membranes. We tested this hypothesis by mutating threonine 276 in cytohesin 2 to aspartic acid to mimic phosphorylation. We found that indeed cytohesin 2 T276D is more strongly associated with membranes than is the wild-type protein (figure 4A). Similar to what we saw with Δcoiled-coil cytohesin 2, a larger fraction of the T276D cytohesin 2 is found in a total membrane fraction than is wild-type cytohesin 2. This supports the hypothesis that phosphorylation of this residue can relieve the auto-inhibition that prevents membrane association of the cytohesins.

**Figure 5:**
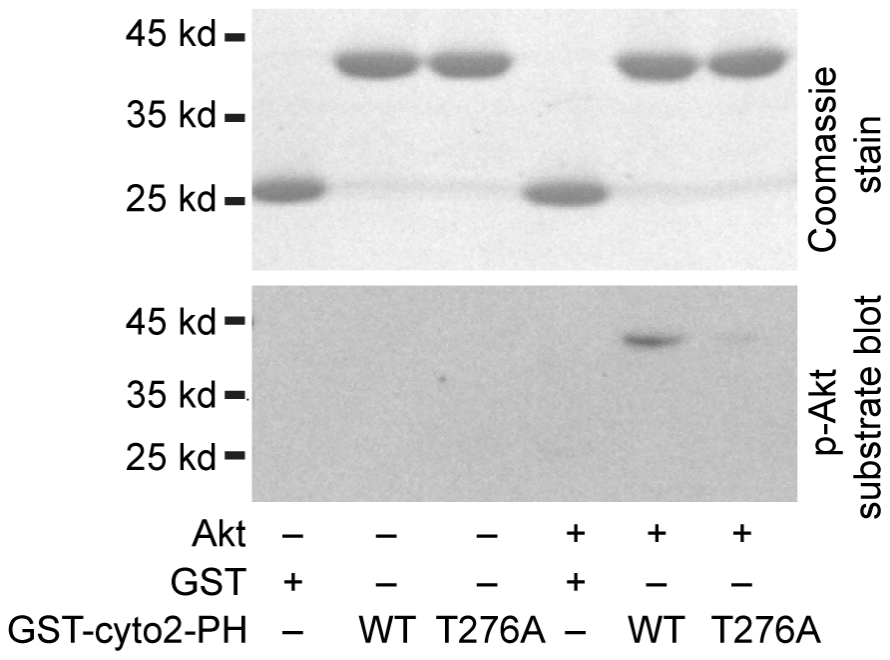
The Cytohesin 2 PH domain is phosphorylated at T276 by Akt. Recombinant GST, GST-cytohesin 2 PH domain, or GST-T276A-cytohesin 2 PH domain were incubated in the presence or absence of recombinant Akt1 as described in Materials and Methods. One tenth of each sample was run out on duplicate gels and either stained with commassie blue or blotted with rabbit anti-Akt substrate antibody.

To further test this hypothesis we created mutations of the T276 residue in Δcoiled-coil cytohesin 2 and tested the ability of these mutations to interact with GST-coiled-coil. Mutation of threonine 276 to aspartic acid (T276D) prevents binding of Δcoiled-coil cytohesin 2 to GST-coiled-coil while mutation of this residue to alanine (T276A) does not (figure 4B). These data support the hypothesis that phosphorylation of this amino acid can disrupt the interaction of the cytohesin coiled-coil domain with the rest of the protein. In order to confirm that this residue can be phosphorylated by Akt, we incubated purified GST-cytohesin 2 PH domain fusions (WT or T276A) with recombinant Akt1 and then blotted them with an antibody that recognizes phosphorylated Akt substrates. After incubation with Akt1, the PH domain of WT but not T276A cytohesin 2 contains phosphorylated Akt substrates (figure 5).

**Figure 6:**
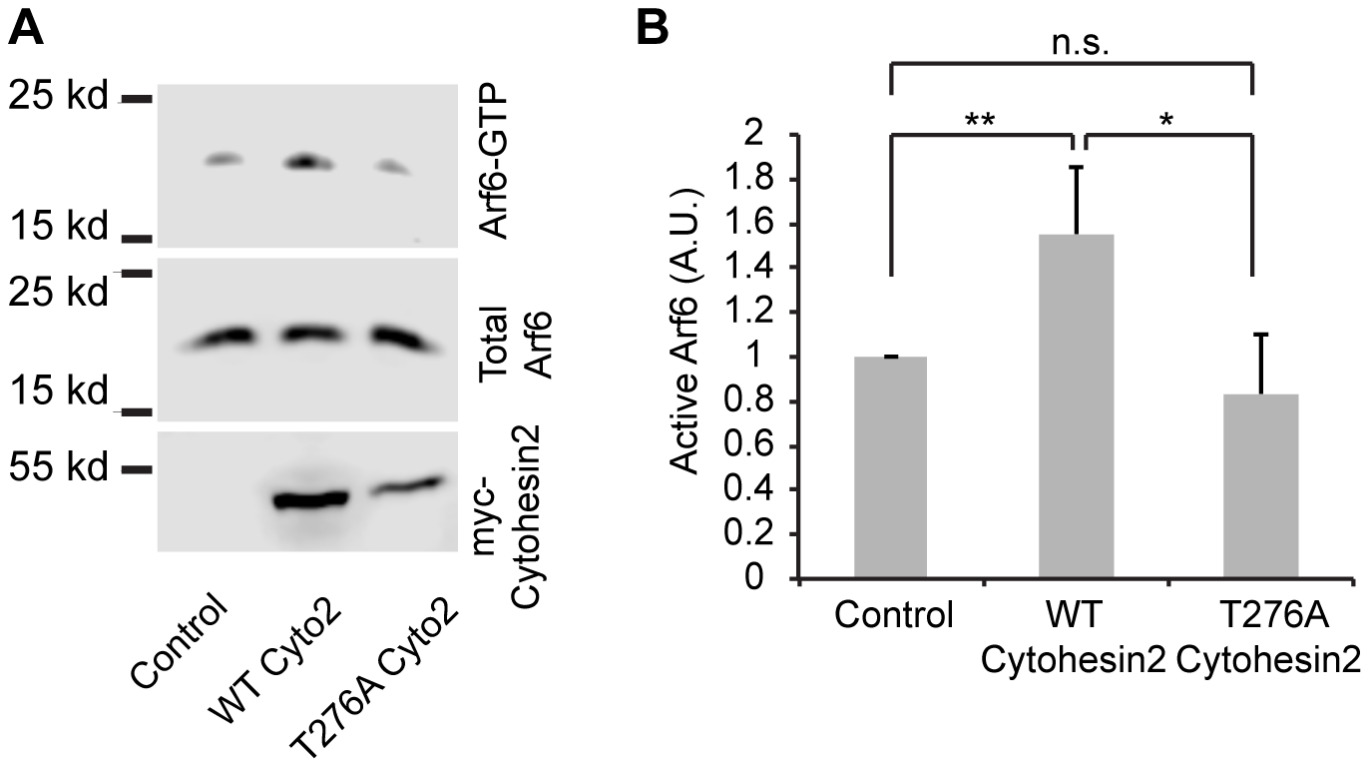
Mutation of Threonine 276 to Alanine inhibits cytohesin 2 activation of Arf6. MDCK cells were transfected with empty vector, cytohesin 2, or cytohesin 2 T276A and active Arf6 was isolated as described in Materials and Methods. A) Representative gel of the pulldown experiments. B) Levels of Arf6 activation in 6 independent pulldown experiments were quantified. Data shown are mean ± standard deviation. The levels of active Arf6 in cytohesin 2 expressing cells were compared to the levels in the vector controls using a paired T-test (N = 6). ** = p<0.01, * = p<0.05, n.s. = no significant difference.

These data suggest that Akt dependent phosphorylation of threonine 276 in cytohesin 2 will allow it to go to membrane surfaces and activate Arfs. Therefore the T276A mutation should reduce the ability of cytohesin 2 to stimulate Arf activation. MDCK cells were transfected with empty vector, WT cytohesin 2, or T276A cytohesin 2 and the levels of active Arf6 were determined using a pulldown assay. Expression of WT but not T276A cytohesin 2 produced a significant increase in the level of active Arf6 in these cells (figure 6).

## Discussion

We have reported here that the cytohesin 2 coiled-coil domain participates in an autoinhibitory interaction with a conserved threonine residue located within the PH domain. This autoinhibitory interaction prevents the association of cytohesin 2 with membranes. A previous study demonstrated that phosphorylation of this residue promoted membrane association, but did not identify a mechanism for this increased membrane localization [12]. We have demonstrated that mutating this threonine to aspartic acid to mimic phosphorylation disrupts the autoinhibitory interaction with the coiled-coil domain, thereby providing a mechanism to explain the enhanced membrane binding.

**Figure 7:**
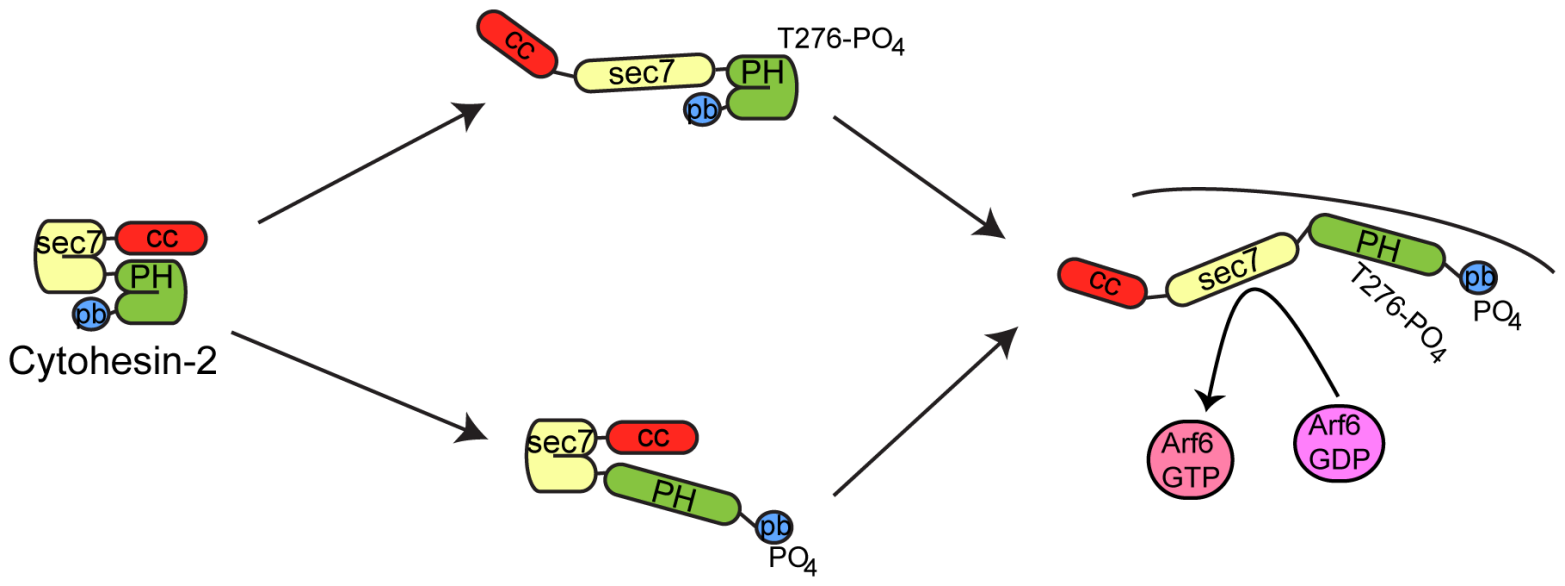
Model for the relief of the two cytohesin autoinhibitory interactions. Full activation of cytohesins will require two separate signals to disrupt both interactions.

The autoinhibitory interaction that we have identified is not the first autoinhibitory interaction identified for the cytohesins. The linker between the Sec7 and PH domain and the C-terminus of the protein form a pseudosubstrate that inhibits the GEF activity of the Sec7 domain [10]. This pseudosubstrate inhibition and the autoinhibitory interaction of the coiled-coil domain appear to be independent negative regulatory intramolecular interactions. The coiled-coil interacts better with the rest of the protein in situations that disrupt the pseudosubstrate interaction (figure 3). Therefore the cytohesins may be acting as signal integrators that require two separate signals to reach full activity (figure 7). Phosphorylation of threonine 276 by Akt would relieve the inhibition of membrane binding by the coiled-coil domain, however in the absence of additional signals the pseudosubstrate interaction would still be inhibiting the catalytic GEF activity (figure 7 upper path). Similarly, phosphorylation of the polybasic domain by PKC or binding of active GTPases to the cytohesin PH domain would relieve the pseudosubstrate inhibition, but lipid binding would still be impaired by the coiled-coil domain (figure 7 lower path). In fact our data suggests that the coiled-coil domain may bind best to the conformation where the pseudosubstrate interaction has been disrupted. Only after receiving signals that disrupt both of these autoinhibitory interactions would full GEF activity and robust Arf activation occur (figure 7 right).

The need for two separate signals to produce a cytohesin with robust GEF activity suggests that the cytohesins have developed to promote Arf activation in response to a stimulus rather than under basal conditions. Cytohesins are also expressed at very low levels in most cells (unpublished observations). Therefore in unstimulated conditions very little Arf is likely to be activated by the cytohesins. Once activated however, cytohesins could produce a burst of active Arf. In support of this idea, the product of cytohesin GEF activity, Arf-GTP, can relieve the pseudosubstrate inhibition. This establishes a positive feedback loop that can produce an explosive increase in active Arf levels once a threshold level of cytohesin GEF activity is obtained [11].

In organisms cell migration is usually initiated through the actions of growth factors [26]–[28]. Arf6 is activated by a number of different growth factors including HGF, PDGF, EGF, CSF and insulin [29]–[33]. Furthermore, cytohesins and cytohesin-binding proteins have been implicated in signal transduction downstream of several of these growth factors [12], [14], [33]–[36]. Cytohesins are well designed to integrate signals from growth factors and other pro-migratory stimuli to promote cell shape changes and cell motility. They may therefore play a central role in initiating movement in response to pro-migratory stimuli.
